# Generation of Novel Chimeric Mice with Humanized Livers by Using Hemizygous cDNA-uPA/SCID Mice

**DOI:** 10.1371/journal.pone.0142145

**Published:** 2015-11-04

**Authors:** Chise Tateno, Yosuke Kawase, Yoshimi Tobita, Satoko Hamamura, Hiroki Ohshita, Hiroshi Yokomichi, Harumi Sanada, Masakazu Kakuni, Akira Shiota, Yuha Kojima, Yuji Ishida, Hiroshi Shitara, Naoko A. Wada, Hiromi Tateishi, Masayuki Sudoh, Shin-ichiro Nagatsuka, Kou-ichi Jishage, Michinori Kohara

**Affiliations:** 1 PhoenixBio Co., Ltd., Higashihiroshima, Hiroshima, Japan; 2 Liver Research Project Center, Hiroshima University, Hiroshima, Japan; 3 Chugai Research Institute for Medical Science, Inc., Gotemba, Shizuoka, Japan; 4 Tokyo Metropolitan Institute of Medical Science, Tokyo, Japan; 5 Chugai Pharmaceutical Co., Ltd., Gotemba, Shizuoka, Japan; 6 Sekisui Medical Co., Ltd., Tokyo, Japan; Centro de Investigación en Medicina Aplicada (CIMA), SPAIN

## Abstract

We have used homozygous albumin enhancer/promoter-driven urokinase-type plasminogen activator/severe combined immunodeficient (uPA/SCID) mice as hosts for chimeric mice with humanized livers. However, uPA/SCID mice show four disadvantages: the human hepatocytes (h-heps) replacement index in mouse liver is decreased due to deletion of uPA transgene by homologous recombination, kidney disorders are likely to develop, body size is small, and hemizygotes cannot be used as hosts as more frequent homologous recombination than homozygotes. To solve these disadvantages, we have established a novel host strain that has a transgene containing albumin promoter/enhancer and urokinase-type plasminogen activator cDNA and has a SCID background (cDNA-uPA/SCID). We applied the embryonic stem cell technique to simultaneously generate a number of transgenic lines, and found the line with the most appropriate levels of uPA expression—not detrimental but with a sufficiently damaged liver. We transplanted h-heps into homozygous and hemizygous cDNA-uPA/SCID mice via the spleen, and monitored their human albumin (h-alb) levels and body weight. Blood h-alb levels and body weight gradually increased in the hemizygous cDNA-uPA/SCID mice and were maintained until they were approximately 30 weeks old. By contrast, blood h-alb levels and body weight in uPA/SCID chimeric mice decreased from 16 weeks of age onwards. A similar decrease in body weight was observed in the homozygous cDNA-uPA/SCID genotype, but h-alb levels were maintained until they were approximately 30 weeks old. Microarray analyses revealed identical h-heps gene expression profiles in homozygous and hemizygous cDNA-uPA/SCID mice were identical to that observed in the uPA/SCID mice. Furthermore, like uPA/SCID chimeric mice, homozygous and hemizygous cDNA-uPA/SCID chimeric mice were successfully infected with hepatitis B virus and C virus. These results indicate that hemizygous cDNA-uPA/SCID mice may be novel and useful hosts for producing chimeric mice for use in future long-term studies, including hepatitis virus infection analysis or drug toxicity studies.

## Introduction

The generation of chimeric mice with humanized livers by transplantation of human hepatocytes (h-heps) into the spleen of urokinase-type plasminogen activator (uPA)/severe combined immunodeficient (SCID) mice has been reported previously [1,2]. We developed a chimeric mouse (PXB-mouse^®^) in which mouse hepatocytes (m-heps) are largely replaced with transplanted h-heps expressing h-cytochrome P450 (CYP) enzymes [2,3], phase II enzymes [4], and transporters [5], and thus have the potential for CYP enzyme induction [2,6,7]. Moreover, human liver chimeric mice that were generated using Fah (−/−)/Rag2 (−/−)/Il2rg (−/−) and TK-NOG mice were able to express h-CYP mRNA in their humanized livers at levels similar to those of h-heps [8,9]. These mice have recently been used as models in drug metabolism and pharmacokinetics (DMPK) and HBV or HCV infection studies to predict human metabolism and drug efficacy in infections caused by hepatitis B virus (HBV) or hepatitis C virus (HCV) [10].

The uPA-transgenic mouse was developed by introducing mouse genomic uPA genes into fertilized eggs [11,12]. However, although the expression of uPA genes initially gave the liver of hemizygotes a pale fatty appearance [11], this was completely replaced 2 months later with red-colored normal m-hep colonies, when the uPA transgenes were deleted by homologous recombination [11]. Because of this replacement, the hemizygotes cannot be used to produce chimeric mice with humanized livers [1]. On the other hand, red colony numbers are reduced in uPA/SCID homozygotes, because independent gene deletion events are required to produce red nodules in mice that carry two genomic copies of the transgene array [11]. Therefore, homozygotes appear to be appropriate hosts for chimeric mice with humanized livers, and uPA/SCID homozygotes have been the most commonly used hosts to generate humanized chimeric mice [10]. However, uPA/SCID mice have the following disadvantages as hosts for chimeric mice: 1) the h-heps replacement index (RI) in mouse liver is decreased, even in homozygotes used in long-term studies, 2) the tendency to develop kidney disorders is increased, 3) body size is decreased [13], and 4) hemizygotes cannot be used as hosts [1]. Despite this, we succeeded in mass-producing chimeric mice with more than a 70% RI using uPA/SCID homozygotes [2]. However, although the RI was consistent until mice were approximately 20 weeks old (about 17 weeks after transplantation), it gradually decreased because of an increase of red m-hep colonies in some mice. Since the number and extent of growth of the red colonies vary, pathological changes of the liver are difficult to determine in long-term toxicological studies or in HCV or HBV drug efficacy studies, because of the variability of pathological features at baseline. Recently, uPA/NOG mice, which were produced by introducing cDNA-uPA genes into fertilized eggs of NOG mice, showed no deletion of transgenes and no growth of normalized mouse colonies in chimeric mice with humanized livers, but only homozygotes could be used to produce them [14].

We hypothesized that the above four disadvantages were caused by inadequate transgene structure and/or very high expression levels of the uPA gene before or after birth. In order to solve these disadvantages, we demonstrated that novel cDNA-uPA transgenic mice could be generated using embryonic stem (ES) cells, and used them to produce novel cDNA-uPA/SCID mice. Using mouse ES cells to generate cDNA-uPA mice, we could obtain a number of transgenic lines simultaneously and could select the best lines to produce chimeric mice with humanized livers. Chimeric mice generated using the cDNA-uPA/SCID mice were larger in body size than those generated using uPA/SCID mice, and did not show deletion of transgenes or kidney disorders. As female uPA/SCID homozygotes are difficult to breed because of reproductive organ atrophy [15], we suggest that if hemizygotes can be used as hosts to transplant h-heps, it will be easier to breed offspring as hosts, and make it possible to generate double transgenic mice by mating them with other genetically-modified mice.

## Materials and Methods

### Ethics statements

All in vivo experiments and protocols for animal experiments were approved by the Laboratory Animal Ethics Committee at PhoenixBio Co., Ltd., Animal Use and Care Committee of the Tokyo Metropolitan Institute of Medical Science, and the Institutional Animal Care and Use Committee at Chugai Pharmaceutical Co., Ltd., respectively.

### Generation of ES cells carrying mouse cDNA-uPA constructs

**Fig 1:**
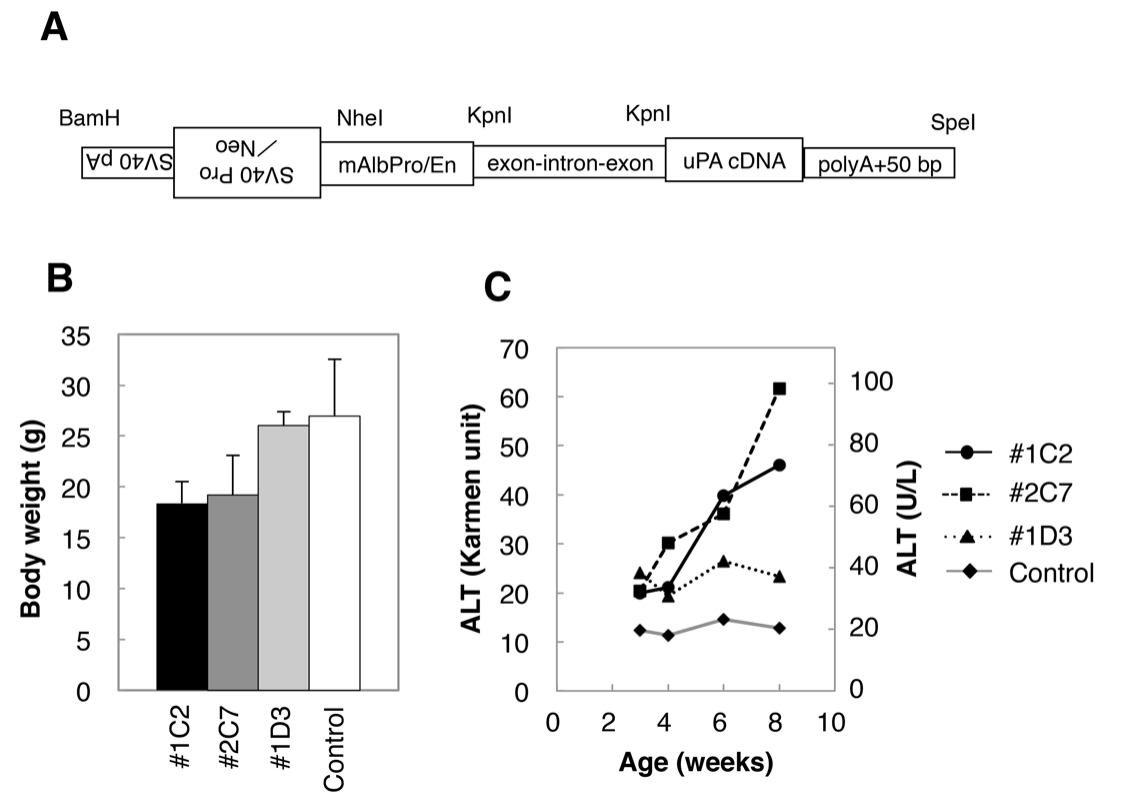
Production of cDNA-uPA mice. (A) Construction of the cDNA-uPA transgene. (B) Body weight in 8-week-old mice and (C) serum ALT activity in #1C2, 2C7, 1D3, and non-transgenic control mice. Body weight of #1C2 and #2C7 mice was lower than that of #1D3 and control mice. ALT activity in #1C2 and #2C7 mice increased from the age of 6–8 weeks. The approximate values of ALT are given on the right side of the vertical axis of the graph. These values were calculated as follows: 1 ALT (IU/L) ≈ 1.58 Karmen.

The expression vector for cDNA-uPA transgenic mice was constructed, as shown in Fig 1A, using mouse cDNA-uPA under the control of murine albumin promoter. The cDNA-uPA vector was introduced by electroporation into ES cells established from 129/SvEv mice. The ES cells were selected in a culture medium containing G418. The transgenic ES cell clones were screened for uPA cDNA by genomic PCR using the forward primer GGGCGGCGGTACCGATCCTGAGAACTTCAGGGTGAG and the reverse primer GGGCGGCGGTACCAATTCTTTGCCAAAATGATGAGA.

### Establishment of cDNA-uPA transgenic mice

The cDNA-uPA transgenic ES cell clones were injected into C57BL/6J mouse blastocysts to produce chimeric mice. Male chimeric mice were crossed with C57BL/6J females to generate offspring. Germline transmission was confirmed by PCR analysis using the primers shown above.

The cDNA-uPA mice (hemizygote, +/wt) were crossed with SCID-beige mice (Taconic Biosciences Inc.,) twice, and their offspring (hemizygote, +/wt) crossed with SCID mice (PhoenixBio, uPA (wt/wt)/SCID (+/+)) twice. cDNA-uPA (+/wt)/SCID (+/+) offspring were intercrossed with each other to obtain cDNA-uPA (+/+)/SCID (+/+) and cDNA-uPA (+/wt)/SCID (+/+) mice. Genotyping to detect the cDNA-uPA transgene was performed by the genomic PCR protocol shown above. To distinguish between cDNA-uPA (+/wt) and cDNA-uPA (+/+) mice, genomic Southern blotting was performed using ^32^P-labeled uPA cDNA fragments (379 bp). Genotyping for SCID mutation was carried out by the PCR-restriction fragment length polymorphism (RFLP) method [2].

### H-heps transplantation

Cryopreserved h-heps were purchased from BD Biosciences (Woburn, MA, USA). BD85 (5-year-old African American girl) and BD195 (2-year-old Hispanic girl) hepatocytes were thawed and transplanted into 2–4-week-old uPA/SCID, cDNA-uPA (+/wt)/SCID, or cDNA-uPA (+/+)/SCID mice via the spleen, under anesthesia [2]. In addition, BD72 (10-year-old Caucasian girl) and BD87 (2-year-old Caucasian boy) hepatocytes were thawed and transplanted into 2–4-week-old uPA/SCID mice. Three weeks after transplantation, blood (2 μL) was collected periodically from the tail vein to measure h-alb concentration. uPA/SCID mice were euthanized at 14 or 28 weeks of age, and hemi- and homozygous cDNA-uPA/SCID mice at 16 or 28 weeks and 17 or 29 weeks of age, respectively.

### uPA and ALT activity levels and h-alb, CRE and BUN concentrations in mouse blood

Blood was isolated from the tail vein of transgenic mice and serum was obtained by centrifugation. To select transgenic mice with liver toxicity due to the uPA transgene, alanine aminotransferase (ALT) activity was measured using the transaminase CII-test kit (WAKO Pure Chemical Industries, Ltd.). uPA and ALT activities in the selected transgenic mice were determined by ELISA (Mouse uPA Activity Assay, Molecular Innovations) and DRI-Chem 3500 (Fujifilm, Tokyo, Japan), respectively.

H-alb concentration in chimeric mouse blood was measured by immunonephelometry in a JEOL BM6050 autoanalyzer (JEOL, Tokyo, Japan) using LX Reagent Eiken Alb II (Eiken Chemical, Tokyo, Japan). Creatinine (CRE) and blood urea nitrogen (BUN) levels were measured by the urease glutamate dehydrogenase method and enzyme method using EKDIA XL ‘Eiken’ CREIII and EKDIA XL ‘Eiken’ UN (Eiken Chemical, Tokyo, Japan), respectively, with a JEOL BM6050 autoanalyzer (JEOL, Tokyo, Japan).

### Histochemistry

Paraffin sections with a thickness of 5 μm were prepared from liver and kidney tissues for hematoxylin and eosin (H&E) staining. Frozen sections (5 μm) were stained with anti-human cytokeratin 8/18 (hCK8/18) mouse monoclonal antibodies (×100 dilution, Cappel Laboratory, Cochranville, PA), specific for h-heps. The h-hep RI was calculated as the ratio of the area occupied by hCK8/18-positive h-heps to the entire area examined on immunohistochemical sections from 7 lobes, as described previously [2]. Frozen sections were also stained using oil red O.

### Microarray analysis

For microarray analysis, total hepatic RNA was extracted from the livers of chimeric BD195- (n = 10), BD72- (n = 3), BD85- (n = 3), and BD87-transplanted (n = 3) uPA/SCID mice and BD195-transplanted cDNA-uPA homo- (n = 3) and hemizygous (n = 3) mice. The liver tissues of uPA/SCID chimeric mice showed three visually identifiable regions of different colors. White and red regions corresponded to original diseased m-hep and uPA gene-deleted m-hep regions, respectively, whereas medium-colored regions, between the white and red regions, corresponded to h-hep areas [16]. The liver tissues of cDNA-uPA/SCID chimeric mice, showed two identical regions of red- and medium-colored areas, which corresponded to m- and h-hep areas. Using a razor blade, h-hep areas from the livers of chimeric mice were dissected, and RNA extraction and microarray analysis were performed. Total RNA was isolated from each h-hep sample using an RNeasy Kit (Qiagen) according to the manufacturer’s instructions. Total RNA concentration and purity was measured using a NanoDrop ND–1000 spectrometer (NanoDrop Technologies Inc., Delaware, USA). RNA integrity was assessed using a Bioanalyzer (Agilent Technologies, California, USA). If total RNA was deemed to be of sufficient quality (A_260_/A_280_ > 1.9 and 28S/18S ratios approaching 2) the sample was stored at −80°C until further analysis. For microarray assays, RNA samples were applied to the GeneChip^®^ Human Genome U-133 Plus 2.0 Array (Affymetrix, Santa Clara, CA, USA) containing 54,675 probe sets according to the manufacturer’s instructions. Gene expression array data were normalized using the MAS5 algorithm (Affymetrix). The signal reliability of each probe was determined based on the MAS5 Call algorithm (Affymetrix), and each probe was assigned to 1 of 3 flags (P: present, M: marginal, and A: absent). To correct for bias between chips, GeneChip CEL files were imported into the Avadis 4.3 data mining and visualization package (Strand Genomics, Bangalore, India). Robust multichip averaging (RMA) was performed as previously described [17]. Donor vs. donor correlation analyses for the RMA-normalized logarithmic expression levels of all or selected (Phase I and Phase II metabolic enzymes) probe sets were performed using the Excel CORREL worksheet function.

### HBV and HCV infection studies

Chimeric 14–15-week-old mice were infected intravenously with 10^4^ HBV or HCV copies. Blood was withdrawn weekly and 2 μL used to measure h-alb concentration, while the remaining blood was used to obtain serum for HBV or HCV analyses. Briefly, individual blood samples were left to coagulate at room temperature for at least 5 min, followed by centrifugation at 13,200 × *g* for 3 min at 4°C.

HBV DNA was extracted from serum using the SMITEST EX-R&D Nucleic Acid Extraction Kit (Medical & Biological Laboratories Co., Ltd., Nagoya, Japan), and dissolved in nuclease-free water (Life Technologies Corporation, Carlsbad, CA, USA). Real-time PCR to measure serum HBV DNA concentration was performed with TaqMan PCR Core Reagents (Life Technologies Corporation) using an ABI Prism 7500 sequence detector system (Life Technologies Corporation). After addition of the PCR reaction mixture to 5 μL of extracted DNA, an initial uracil-N-glycosylase activation at 50°C for 2 min was followed by inactivation at 95°C for 10 min. Subsequent PCR amplification consisted of 53 cycles of denaturation at 95°C for 20 s, and annealing and extension at 60°C for 1 min per cycle using an ABI 7500 sequence detector. The average serum HBV DNA level was calculated from the values of the two separate wells. Primers and probes consisted of the following: Forward primer HB-166-S21 (nucleotides 166–186), 5’-CACATCAGGATTCCTAGGACC-3’, Reverse primer HB-344-R20 (nucleotides 344–325), 5’-AGGTTGGTGAGTGATTGGAG-3’, TaqMan probe HB-242-S26FT (nucleotides 242–267), 5’-CAGAGTCTAGACTCGTGGTGGACTTC-3’ (Dye: FAM for 5’, TAMRA for 3’) [18]. The lowest quantification limit of this assay was < 2.0 × 10^4^ copies/mL.

HCV RNA was extracted from serum using the SepaGene RV-R (Eidia Co., Ltd., Tokyo, Japan), and dissolved in nuclease-free water (Life Technologies Corporation, Carlsbad, CA, USA) containing 1 mM DTT (Promega Corporation, Tokyo, Japan) and 0.4 U/μL ribonuclease inhibitor (Takara Bio Inc., Shiga, Japan). Real-time PCR to measure serum HCV RNA concentration was performed with TaqMan PCR Core Reagents (Life Technologies Corporation) using an ABI Prism 7500 sequence detector system (Life Technologies Corporation). After addition of PCR reaction mixture to 2.5 μL of extracted RNA, an initial uracil-N-glycosylase activation at 50°C for 2 min was followed by inactivation at 95°C for 5 min. Subsequent PCR amplification consisted of 50 cycles of denaturation at 95°C for 20 s, and annealing and extension at 60°C for 1 min per cycle in an ABI 7500 sequence detector. The average serum HCV RNA level was calculated from the values of the two separate wells. Primers and probes consisted of the following: Forward primer R6-130-S17 (nucleotides 130–146), 5’-CGGGAGAGCCATAGTGG-3’, Reverse primer R6-290-R19 (nucleotides 290–272), 5’-AGTACCACAAGGCCTTTCG -3’, TaqMan probe R6-148-S21FT (nucleotides 148–168), 5’-CTGCGGAACCGGTGAGTACAC -3’ (Dye: FAM for 5’, TAMRA for 3’) [19]. The lowest quantification limit of this assay was <4.0 × 10^4^ copies/mL.

## Results

### Generation of cDNA-uPA transgenic mouse

We established 93 ES cell clones carrying the cDNA-uPA transgene after G418 selection and PCR screening, and injected them into C57BL/6J blastocysts to produce chimeric mice. The male chimeric mice obtained from 32 ES cell clones were bred with C57BL/6J females to establish the cDNA-uPA transgenic mice. As a result, we obtained 14 lines of cDNA-uPA transgenic mice. Of them, 3 lines of mice (#1C2, #2C7 and #1D3, which have been named B6;129S6-Tg(Alb-uPA)1Csk, B6;129S6-Tg(Alb-uPA)2Csk, B6;129S6-Tg(Alb-uPA)3Csk, respectively) showed an elevated serum ALT activity above that of non-transgenic mice. Body weight of the #1C2 and #2C7 mice was lower than that of the control and #1D3 mice (Fig 1B). ALT levels increased from 6–8 weeks in #1C2 and #2C7 mice, whereas those in #1D3 did not (Fig 1C). Two lines with high serum ALT levels (#1C2 and #2C7) were selected, and backcrossed with SCID-beige mice and SCID mice, and crossed with each other, to obtain hemizygotes and homozygotes.

### H-alb concentration in chimeric mice

**Fig 2:**
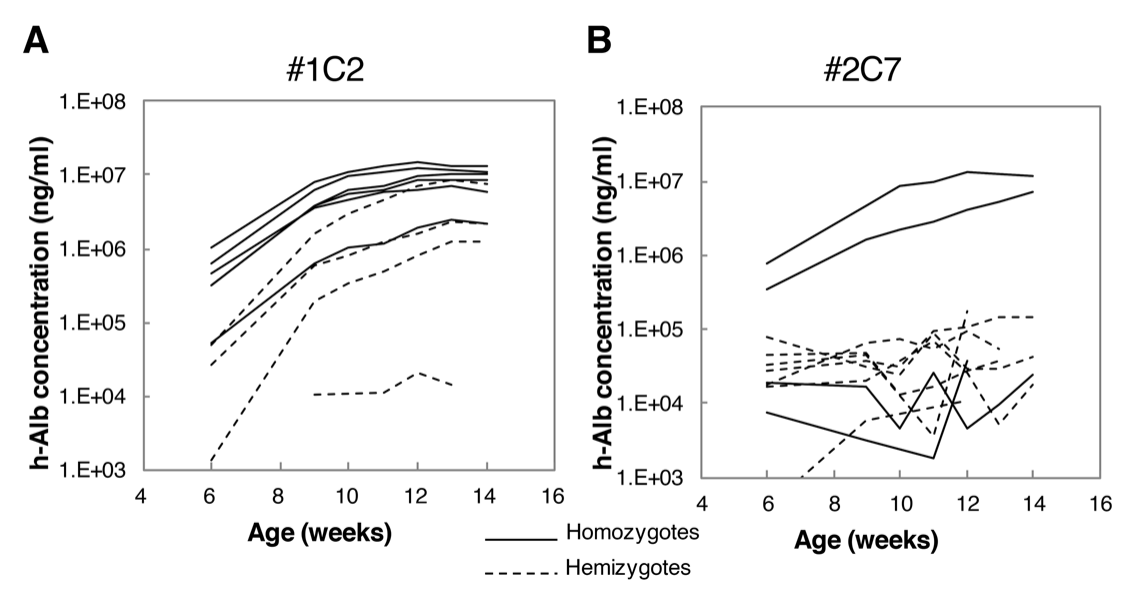
Changes in blood h-alb concentrations. BD85 h-heps were transplanted into (A) #1C2 and (B) #2C7 hemi and homozygotes. Solid and dotted lines show homozygotes and hemizygotes, respectively.

H-heps (BD85, 2.5 × 10^5^ cells) were transplanted into 2–4-week-old #1C2 (homo: 7 mice, hemi: 4 mice) and #2C7 (homo: 4 mice, hemi: 7 mice) mice via the spleen. Serum h-alb levels of #1C2 and #2C7 mice are shown in Fig 2A and 2B, respectively. One #1C2 hemizygous mouse died at 13 weeks of age. By 6 weeks of age (3 weeks after transplantation), h-alb levels were higher in #1C2 homozygotes than in hemizygotes, reaching >7 mg/mL in 4 of 5 homozygotes. By 14 weeks of age, 1 hemizygote reached >7 mg/mL, but 2 hemizygotes still showed h-alb levels <7 mg/mL (Fig 2A), and the levels of h-alb in 2 of 4 #2C7 homozygotes reached >7 mg/ml, but no increase was observed in any of the 7 hemizygotes (Fig 2B). Independent transplantation of 2.5 × 10^5^ BD85 h-hep donor cells into 28 #1C2 and 18 #2C7 homozygotes and 28 #1C2 and 15 #2C7 hemizygotes resulted in h-alb levels >7 mg/mL in 61%, 36%, 28%, and 0% of mice, respectively. Therefore, #1C2 homo- and hemizygotes were used for further studies.

### uPA and ALT activity in non-transplanted uPA/SCID and #1C2 mice

**Fig 3:**
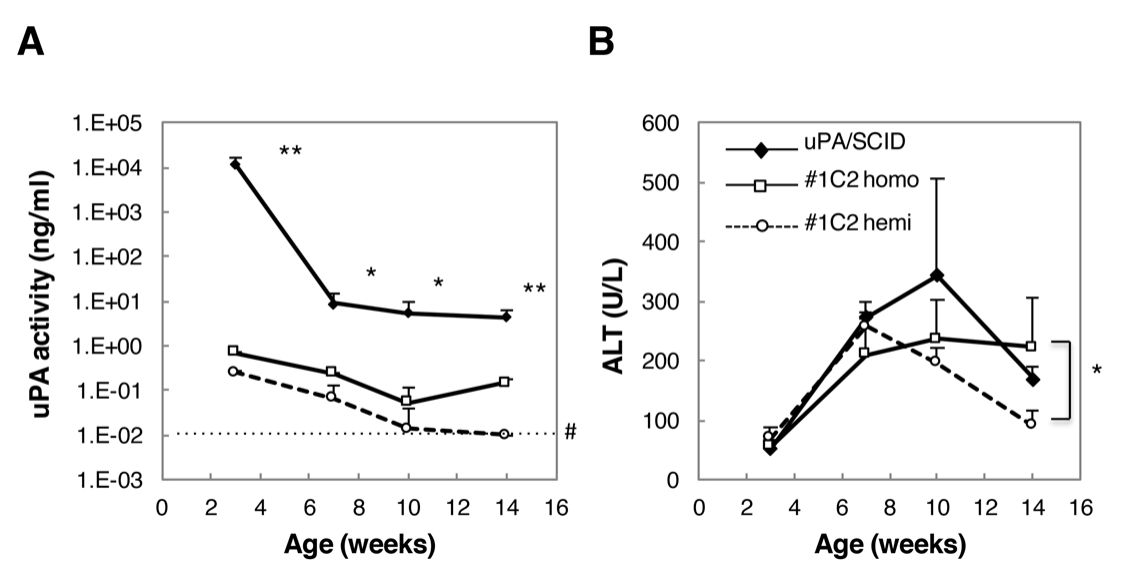
uPA and ALT activity in uPA/SCID mice and #1C2 homo- and hemizygotes. (A) uPA activity was more than 100 times higher in 14-week-old uPA/SCID mice than #1C2 mice. #1C2 homozygotes showed higher uPA activity than hemizygotes. (B) No statistical differences in ALT activity among the three groups at 3, 7, and 10 weeks old were observed. Fourteen-week-old #1C2 homozygotes showed ALT activity that was approximately 2 times higher than that of #1C2 hemizygotes. *, p < 0.05; **, p < 0.01; #, measurement limit.

uPA and ALT levels of activity were measured in sera obtained from male uPA/SCID #1C2 homozygotes and hemizygotes. By 3 weeks of age, serum uPA activity was approximately 10^4^ times higher in uPA/SCID mice than in #1C2 homozygotes, and decreased thereafter, but remained approximately 10^2^ times higher than in #1C2 homozygotes at 14 weeks of age (Fig 3A). Similarly, uPA activity in #1C2 hemiozygotes was approximately 4 times lower than in homozygotes at 3 weeks of age, and decreased gradually thereafter (Fig 3A).

By contrast, ALT activity in uPA/SCID mouse sera did not differ between groups as much as uPA activity did (Fig 3B). In fact, ALT activity was similar between uPA/SCID and #1C2 homo- and hemizygotes in 3- and 7-week-old mice, but in 14-week-old #1C2 hemizygotes, ALT activity was approximately 100 U/L lower than in uPA/SCID and #1C2 homozygotes (~200 U/L).

### Gross appearance and histopathology in non-transplanted uPA/SCID and #1C2 mouse livers

Although livers of non-transplanted 3-week-old uPA/SCID and #1C2 mice were whitish, livers of 14-week-old uPA/SCID mice showed white and red areas derived from diseased and transgene deletion–normalized m-hep areas, respectively. However, #1C2 homo- and hemizygous livers from 14-week-old mice were red-colored.

**Fig 4:**
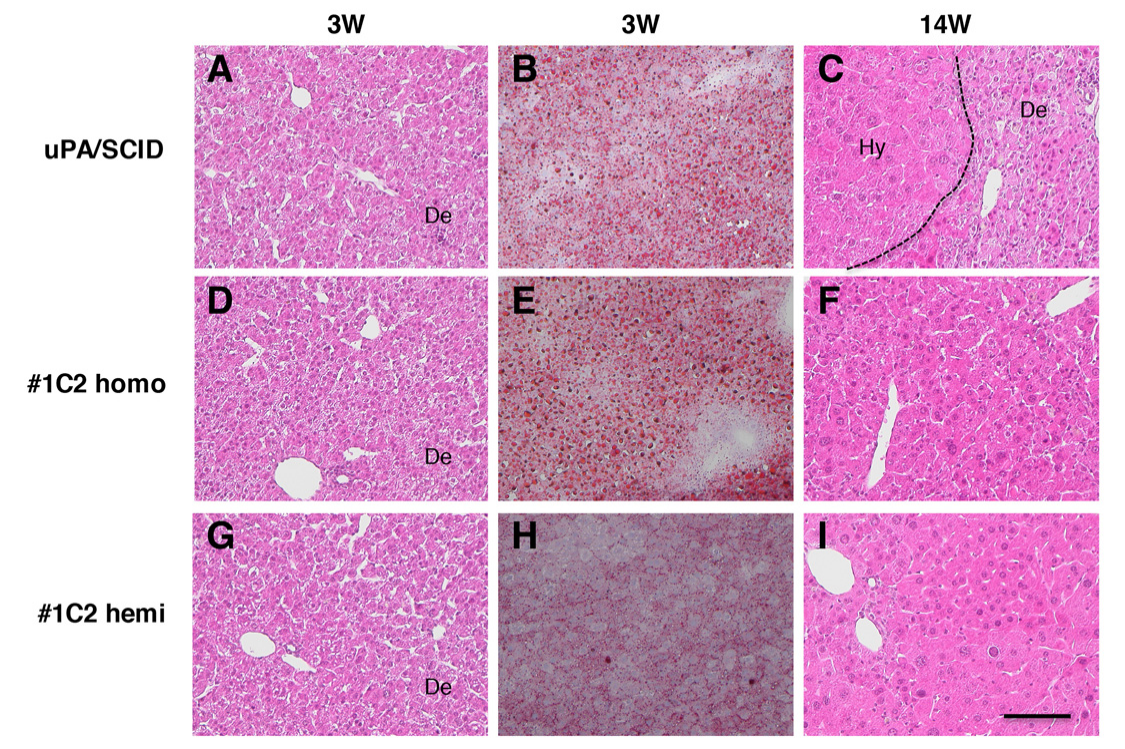
Histological findings in uPA/SCID and #1C2 homo- and hemizygous livers. H&E staining of (A and C) uPA/SCID, (D and F) #1C2 homozygous and (G and I) #1C2 hemizygous mice aged 3 and 14 weeks, respectively. Oil red O staining in 3-week-old (B) uPA/SCID and #1C2 (E) homo and (H) hemizygous mouse livers. Hepatocytes of all 3 groups were degenerating in 3-week-old mice. In 14-week-old mice, degenerating hepatocytes and hyperplastic m-hep colonies were observed in uPA/SCID mice but not in #1C2 homo- and hemizygotes. Varying m-hep sizes were observed in 3-week-old #1C2 homo- and hemizygotes. Lipid droplets were more abundant in uPA/SCID mice and #1C2 homozygotes than #1C2 hemizygotes. De, degenerating hepatocytes. Hy, hyperplastic hepatocyte colony. Bar, 100 μm.

Histopathological examination of H&E stained m-hep sections in 3-week-old uPA/SCID and #1C2 homo- and hemizygous mice showed a similar atrophic morphology (Fig 4A, 4D and 4G), with small lipid droplets seen after oil red O staining (Fig 4B, 4E and 4H). These were particularly abundant in uPA/SCID and #1C2 homozygous mouse livers (Fig 4B and 4E), but smaller in size and quantity in #1C2 hemizygous livers (Fig 4H). Although, the livers of 14-week-old uPA/SCID mice showed atrophic and m-hep hyperplastic colony areas (Fig 4C), the livers in #1C2 homo- and hemizygotes showed no m-hep colonies, but nuclei and hepatocytes of varying sizes were observed (Fig 4F and 4I).

### Repopulation of mouse livers with h-heps

**Fig 5:**
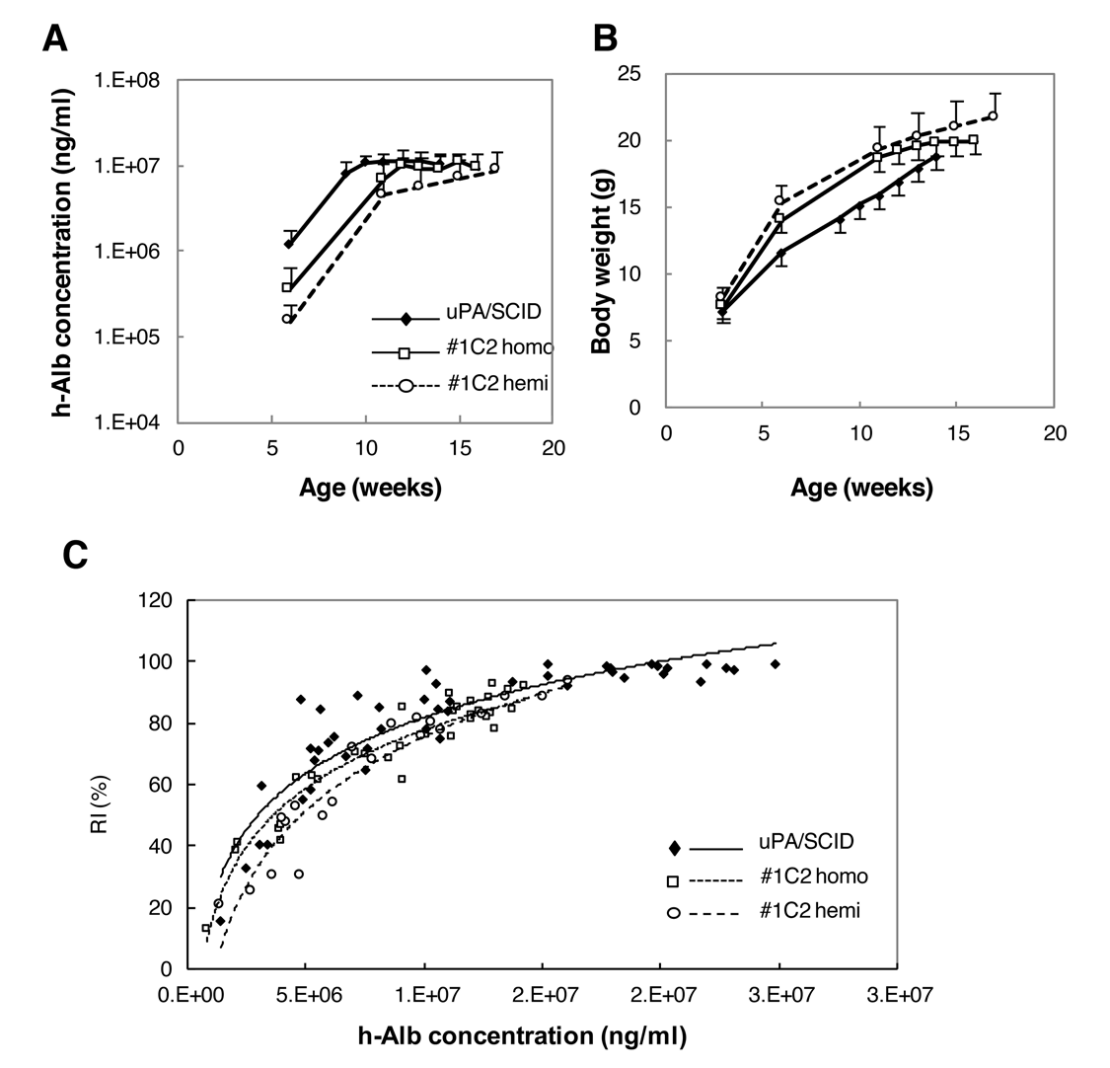
Changes in h-alb concentration and body weight in chimeric mice up to 4 months of age. (A) h-alb levels in uPA/SCID chimeric mice were the highest among the 3 types of chimeric mice, and those of #1C2 homozygous mice were higher than those of hemizygous mice by 6 weeks of age. H-alb levels in uPA/SCID and #1C2 homozygous mice reached a plateau by 10 and 11 weeks of age, respectively, whereas those in #1C2 hemizygous mice continued to increase up to 17 weeks of age. (B) Body weight of #1C2 hemizygous mice was the highest, followed by #1C2 homozygous mice, and then uPA/SCID chimeric mice. (C) RI and h-alb concentrations plots showed similar correlation curves in uPA/SCID and #1C2 homo- and hemizygous chimeric mice.

Following transplantation of BD195 h-heps into uPA/SCID (1.25 × 10^5^ cells/mouse), #1C2 homozygous (1.25 × 10^5^ cells/mouse), and #1C2 hemizygous males (2.5 × 10^5^ cells/mouse), 100 uPA/SCID, 100 #1C2 homozygous, and 51 #1C2 hemizygous males were alive after 14, 16, and 17 weeks, respectively. Their h-alb levels and body weight were monitored and are shown in Fig 5A and 5B and Table 1. The survival rate of uPA/SCID, #1C2 homozygote, and #1C2 hemizygous chimeric mice was 74%, 99%, and 100%, respectively. The engraftment of h-heps into mouse livers was different in uPA/SCID, #1C2 homozygotes, and #1C2 hemizygotes. The levels of h-alb were higher in 6-week-old uPA/SCID mice (3 weeks after transplantation) than in #1C2 homozygotes, and lowest in #1C2 hemizygotes (Fig 5A). The rate of increase in h-alb level was almost similar in all groups (Fig 5A), and reached a plateau in 10- and 11-week-old uPA/SCID and #1C2 homozygous mice, respectively. Although h-alb levels in #1C2 hemizygotes were lower than in the other groups, they continued to increase up to at least 17 weeks after birth. The body weight at over 6 weeks old was higher in #1C2 hemizygotes than in #1C2 homozygotes, and lowest in uPA/SCID mice (Fig 5B, Table 1).

**Table 1:** Blood h-alb levels and body weight in 14–17-week-old host mice.

Host mouse	sex	n	Age	Number of	h-Alb	Body weight
			(weeks)	transplanted cells	(mg/ml)	(g)
uPA/SCID	M	100	14	1.25 × 10^5^	10.2 ± 3.6	18.8 ± 3.6
#1C2 homo	M	100	15	1.25 ×10^5^	11.1 ± 2.4	19.8 ± 2.5
			16		9.2 ± 4.4	19.9 ± 2.7
#1C2 hemi	M	51	15	2.50 ×10^5^	7.1 ± 4.6	21.0 ± 1.8
			17		8.6 ± 5.5	21.7 ± 1.8

BD195 hepatocytes were used as donor cells.

RIs were determined by examining hCK8/18 immunostained liver sections of 44 uPA/SCID, 32 #1C2 homozygotes, and 20 #1C2 hemizygotes, and h-alb concentrations and RIs from male and female chimeric mice were plotted as shown in Fig 5C. Levels of h-alb and RIs correlated well in uPA/SCID mice, as previously shown [2]. Moreover, h-alb levels and RI also correlated in #1C2 homo- and hemizygotes, which showed similar correlation curves (Fig 5C). The R-squared values of the trend lines were 0.83, 0.92, and 0.90 in uPA/SCID, #1C2 homozygotes, and #1C2 hemizygotes, respectively.

### Histopathology and kidney function measurements of uPA/SCID and #1C2 homo- and hemizygous chimeric mouse livers

**Fig 6:**
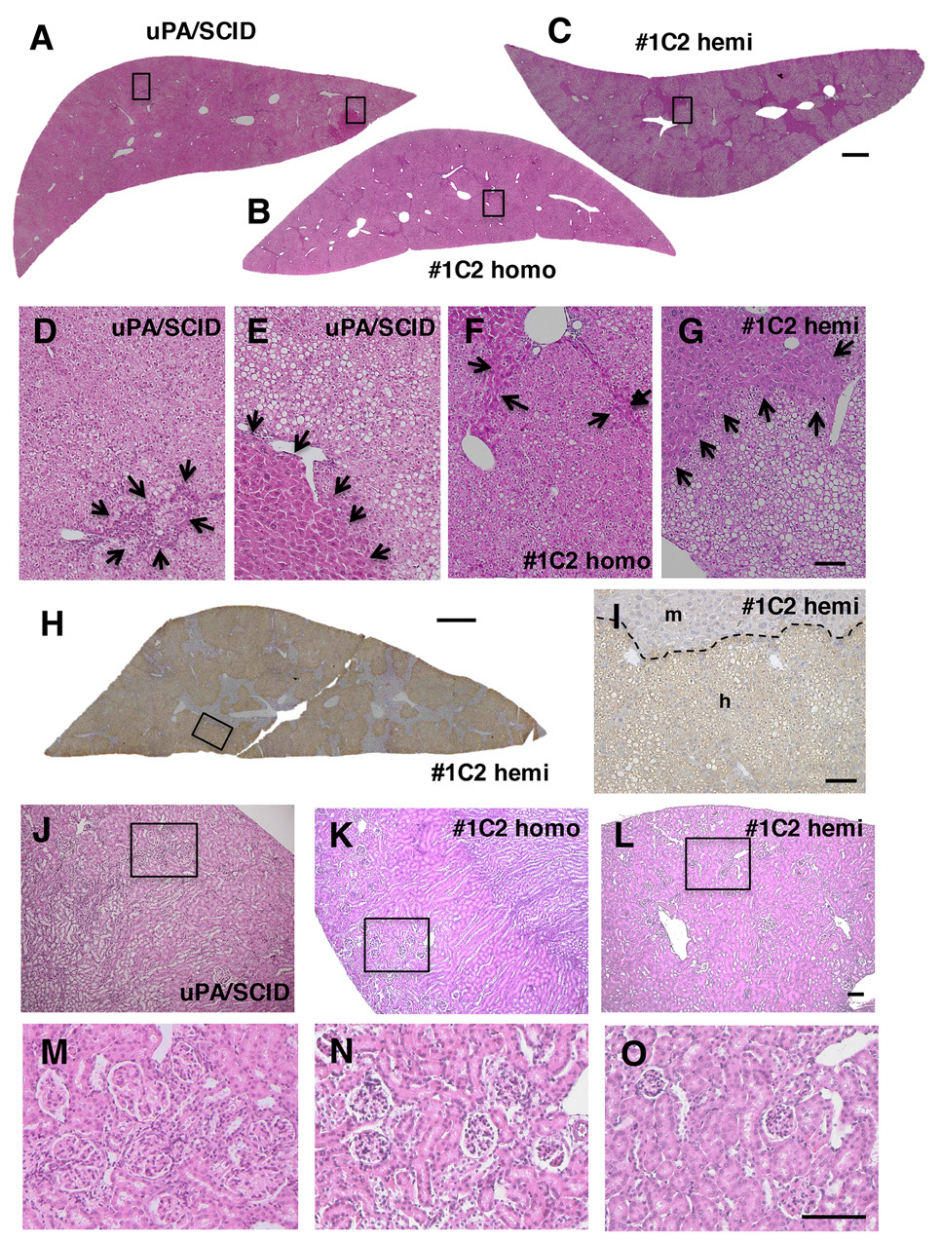
Histopathological findings in chimeric mouse livers and kidneys. H&E staining of left lateral lobes of (A) uPA/SCID chimeric mice, (B) #1C2 homozygous and (C) #1C2 hemizygous chimeric mice, and magnifications of the rectangular parts (D) on the left side of A and (E) on the right side of A, (F) in B, and (G) in C are shown. h-heps with a clear cytoplasm and lipid droplets occupied most areas of the liver sections (A-G). Arrows show (D) degenerating m-heps and (E) hyperplastic m-hep nodules in uPA/SCID chimeric mice. M-heps with eosinophilic cytoplasm of various sizes are shown by arrows in (F) #1C2 homozygous and (G) hemizygous chimeric mice. (H) The left lateral lobe of a #1C2 hemizygous chimeric mouse was immunostained with anti-hCK8/18 antibodies. H-heps were brown-colored, and (I) an area of rectangle was magnified. m, m-heps, h, h-heps. Kidney sections in (J, M) uPA/SCID, (K, N) #1C2 homozygote and (L, O) hemizygous chimeric mice were stained with H&E. Enlarged glomeruli and glomerulosclerosis were observed in uPA/SCID mice (J, M). No pathological findings were observed in (K, N) #1C2 homozygote and (L, O) hemizygous chimeric mouse kidneys. (M), (N) and (O) were high magnification of (J), (K) and (L), respectively. Bar, 100 μm in D-G, I and J-O. Bar, 1 mm in A-C and H.

H&E stained sections of chimeric mouse livers from uPA/SCID and #1C2 homo- and hemizygotes were examined for gross pathology and histopathology. Gross pathologic findings in uPA/SCID chimeric mice included highly repopulated (RI > 70%) white, red, and medium-colored areas, and round-shaped colonies in red-colored areas, as previously shown [2]. By contrast, although medium-colored areas and red irregular areas were observed in chimeric livers from #1C2 homo- and hemizygotes, red colonies were absent. Moreover, H&E stained sections showed that most highly repopulated areas in chimeric mice produced by the 3 types of host mice were occupied with h-heps showing a clear cytoplasm (Fig 6A–6G). Furthermore, these areas were stained with anti-hCK8/18 antibodies (Fig 6H and 6I) [2]. Chimeric mouse livers from uPA/SCID mice showed atrophic hepatocyte areas (Fig 6D) and normal mouse hepatocyte colonies that resulted from uPA-transgenes being deleted (Fig 6E), while eosinophilic areas composed of m-heps of varying sizes were observed in #1C2 homo- and hemizygous chimeric mouse livers (Fig 6F and 6G). Chimeric uPA/SCID mouse kidneys reportedly show some kind of disorder [2]. In fact, we observed glomerulosclerosis in H&E stained kidney sections not only in our uPA/SCID chimeric mice (Fig 6J), but also in non-transplanted uPA/SCID host mice. However, kidneys were normal in #1C2 homo- and hemizygous chimeric mice (Fig 6K and 6L). In addition, CRE and BUN levels were measured in 17- or 18-week-old uPA/SCID chimeric mice (n = 11) and #1C2 hemizygous chimeric mice (n = 14). CRE levels in uPA/SCID chimeric mice and #1C2 hemizygous chimeric mice were found to be 0.13 ± 0.20 and 0.10 ± 0.01 mg/dl, respectively. BUN levels in uPA/SCID chimeric mice and #1C2 hemizygous chimeric mice were found to be 39.5 ± 49.6 and 28.2 ± 7.9 mg/dl, respectively. Both CRE and BUN levels were higher and had wider ranges in uPA/SCID chimeric mice than in #1C2 hemizygous chimeric mice. Therefore, kidney function was improved in #1C2 hemizygous chimeric mice than in uPA/SCID chimeric mice.

### Long-term observation of chimeric mice

**Fig 7:**
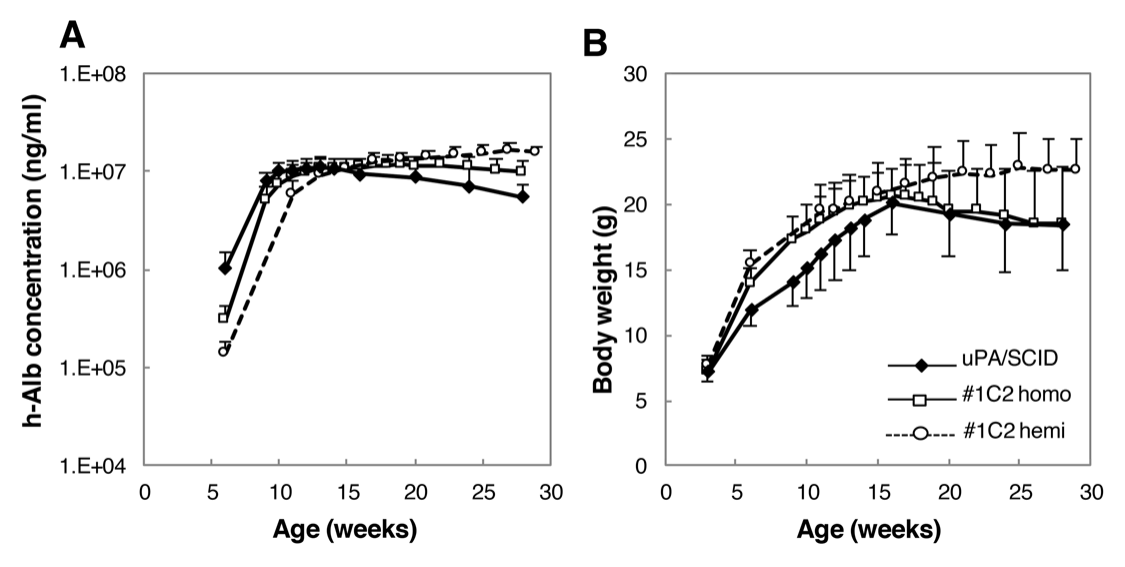
Changes in h-alb levels and body weight of chimeric mice during a long-term period. 14-week-old chimeric mice with >70% RI were selected based on h-alb levels and observed until they were 28 or 29 weeks old. (A) After 16 weeks of age, h-alb levels in uPA/SCID chimeric mice gradually decreased, whereas those of #1C2 homozygous chimeric mice stabilized, but those of #1C2 hemizygous chimeric mice increased gradually until mice were at least 28 weeks old. (B) Body weight of uPA/SCID and #1C2 homozygous chimeric mice decreased gradually after 16 weeks of age, but increased in #1C2 homo chimeras until mice were 18 weeks old, and then stabilized.

Chimeric 14-week-old uPA/SCID and #1C2 homo- and hemizygous mice with h-alb blood levels >7 mg/mL were selected and observed until they were 28–29 weeks old. Blood levels of h-alb in uPA/SCID chimeric mice decreased gradually from approximately 16 weeks onwards because of the growth of transgene-deleted normalized m-heps. H-alb blood levels and body weight of 3 groups until around 16 week old in this long-term study were reproducible in the short-term study (Fig 5). However, while #1C2 homo- and hemizygous chimeric mice were able to maintain their h-alb levels until they were 20 and 29 weeks old, respectively, h-alb levels decreased slightly thereafter in #1C2 homozygotes (Fig 7A).

Body weight increased in parallel in #1C2 homo- and hemizygous chimeric mice, but was lower in 13-week-old uPA/SCID mice. However, the body weight of uPA/SCID and #1C2 homozygous chimeric mice decreased gradually and at a similar rate from 16 weeks of age onwards. On the other hand, the body weight of #1C2 hemizygous chimeric mice continuously increased until they were 20 weeks old and stabilized thereafter (Fig 7B). By the time mice were 28–29 weeks old, the mean body weight in #1C2 hemizygotes was 4 g higher than in uPA/SCID and #1C2 homozygous chimeric mice (Table 2). Survival rates of 28-week-old uPA/SCID and #1C2 homo- and hemizygous chimeric mice were 58.8%, 71.4%, and 100%, respectively (Table 2).

**Table 2:** Mouse blood h-Alb levels and body weight 28–29 weeks after birth.

Host mouse	sex	n*^1^	Age	Number of	h-Alb	Body weight	Survival rate at
			(weeks)	transplanted cells	(mg/ml)	(g)	28-week-old (%)
uPA/SCID	M	34	28	2.5 ×10^5^	5.5 ± 1.9 (20)*^2^	18.4 ± 3.4 (20)	58.8
#1C2 homo	M	28	28	1.25 ×10^5^	9.8 ± 3.4 (20)	18.5 ± 4.3 (20)	71.4
#1C2 hemi	M	27	29	1.25–4.0 ×10^5^	15.7± 2.2 (24)	22.6 ± 2.31 (24)	100

BD85 and BD195 hepatocytes were used as donors for uPA/SCID mice, and #1C2 homo- and hemizygotes, respectively.

*^1^ Number of animals at the start point of the study.

*^2^ Number in parenthesis shows number of animals which was alive at the final point of the study.

### mRNA expression in the livers of uPA/SCID and #1C2 homo- and hemizygous chimeric mice

RNA was extracted from chimeric mouse liver tissues of BD195- (n = 10), BD72- (n = 3), BD85- (n = 3), and BD87-transplanted (n = 3) uPA/SCID mice and from BD195-transplanted cDNA-uPA homo- (n = 3) and hemizygous (n = 3) mice for microarray analyses (Gene Expression Omnibus, Accession No. GSE69936). Results of correlation tests are shown in Table 3. Correlation coefficients between uPA/SCID mice transplanted with h-heps from different donors ranged from 0.955 to 0.984, while those between different host mice transplanted with h-heps from the same donor (BD195) ranged from 0.985 to 0.997. Similarly, correlation coefficients between different host mice transplanted with h-heps from different donors ranged from 0.946 to 0.964 (Table 3). Based on these results, we confirmed that the gene expression in h-heps (BD195) transplanted into uPA/SCID and #1C2 homo- and hemizygous chimeric mice was identical.

**Table 3:** Correlation coefficients between each chimeric mouse group.

	1	2	3	4	5	6
Donor	BD195	BD72	BD85	BD87	BD195	BD195
N	10	3	3	3	3	3
Sex	M	M	M	M	M	M
Gene	uPA/SCID	uPA/SCID	uPA/SCID	uPA/SCID	#1C2	#1C2
homo/hemi	homo	homo	homo	homo	homo	hemi
	1	2	3	4	5	6
1		0.967	0.955	0.971	0.989	0.985
2			0.976	0.984	0.964	0.960
3				0.983	0.948	0.946
4					0.964	0.961
5						0.997

**Fig 8:**
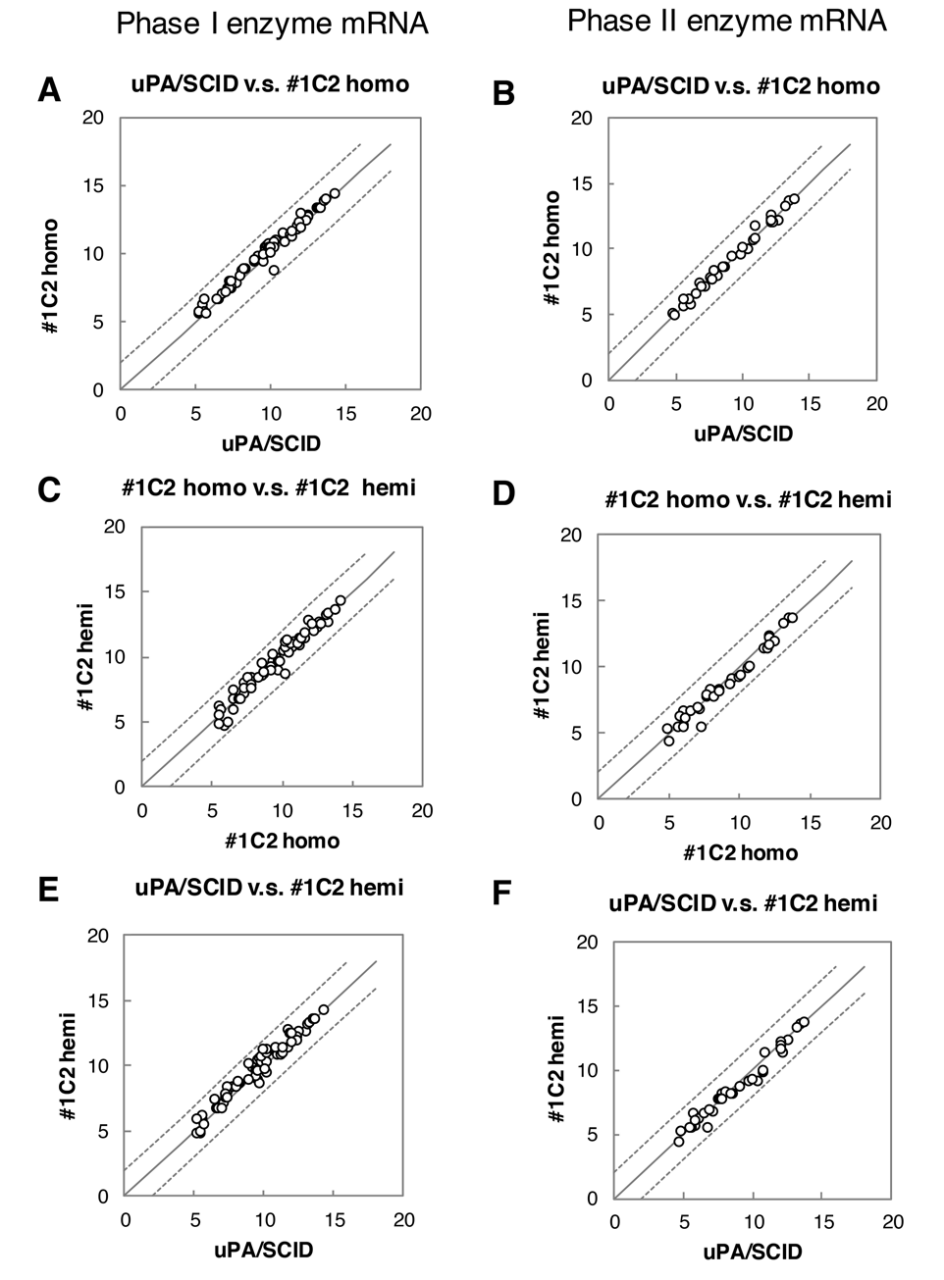
Microarray mRNA expression analysis of phase I and II metabolic enzymes levels in chimeric mice. mRNA expression levels of phase I and II expressed in human livers in (A and B) uPA/SCID and #1C2 homozygous mice, (C and D) #1C2 homo- and hemizygous mice, and (E and F) uPA/SCID and #1C2 mice were plotted. Phase I metabolic enzymes are CYP1A1, 1A2, 1B1, 2A6, 26A1, 2B6, 2C8, 2C9, 2C18, 2C19, 2D6, 2E1, 2J2, 3A4, 3A5, 3A7, 3A43, 39A1, 4A11, 4F2, 4F3, 7A1, 7B1, 8B1. Phase II metabolic enzymes are UGT1A6, 2A1, 2B15, 2B17, 2B4, SULT1A1, 1A3, 1B1, 1E1, 2A1, GSTA1, A4, M1, M2, M3, M4, P1, T1, Z1, and NAT1, 2. The solid and dotted lines represent unity and 4-fold differences, respectively. All values were within the 4-fold difference range.

Expression levels of phase I [Cytochrome P450s (CYPs)] [20,21] and phase II [UDP-glucuronosyltransferases (UGTs), sulfotransferase (SULTs), glutathione S-transferase (GSTs) and N-acetyltransferase (NATs)] [21,22] liver drug-metabolizing enzyme mRNAs were plotted on correlation graphs as shown in Fig 8. Expression levels in different host chimeric mice that were transplanted with h-heps from the same donor were similar and within 4-fold difference.

### HBV and HCV susceptibility in uPA/SCID and #1C2 homo- and hemizygous chimeric mice

**Fig 9:**
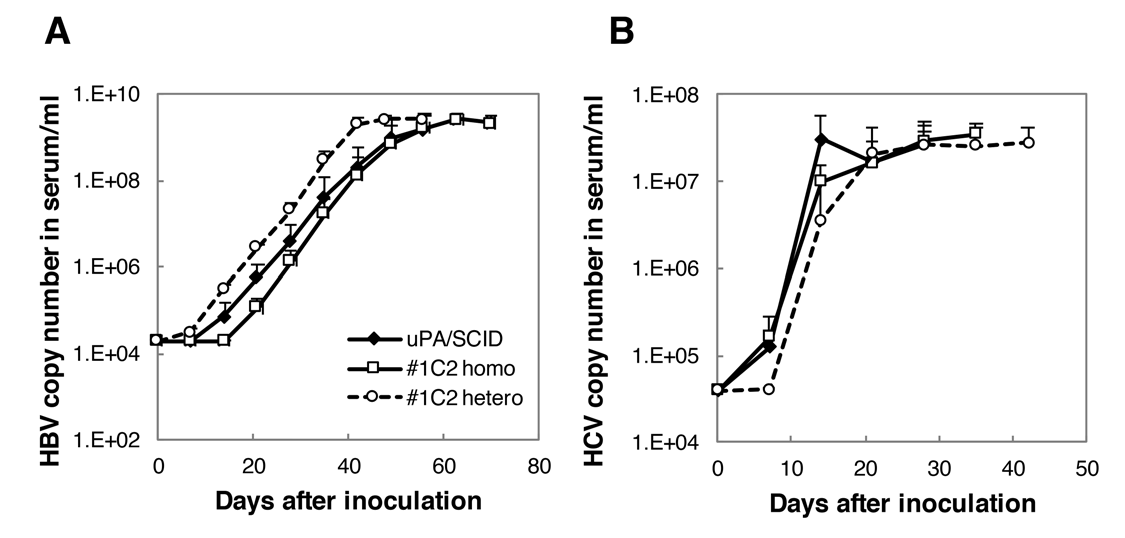
HBV and HCV infections in chimeric mice. Chimeric mice shown in Table 4 were inoculated with (A) HBV and (B) HCV, and serum HBV and HCV copy numbers were monitored up to 70 days and 40 days post-infection, respectively. HBV and HCV copy numbers did not differ among groups and increased up to approximately 50 and 20 days post-infection, respectively, reaching a plateau afterwards.

uPA/SCID and #1C2 homo and hemizygous chimeric mice were inoculated with HBV or HCV. HBV DNA and HCV RNA copy numbers increased and reached a plateau 40–50 days and 28 days after inoculation, respectively, with HCV RNA copy numbers increasing more rapidly than those of HBV in all groups (Fig 9). HBV copy number in serum was higher in #1C2 homozygous chimeric mice than that in uPA/SCID or #1C2 heterozygous chimeric mice until around 40 days after inoculation, but plateau levels of them were similar (Fig 9A).

**Table 4:** Information of animals infected with HBV or HCV.

Strain	Sex	n	Age (weeks)	h-Alb (mg/ml)	Expected RI(%)	Body weight (g)
HBV Infection						
uPA/SCID	M	4	13	8.6 ± 2.6	78.0 ± 7.2	17.6 ± 2.1
#1C2 homo	M	5	11	11.8 ± 2.8	80.1 ± 8.3	18.9 ± 1.3
#1C2 hemi	M	5	14	10.1 ± 1.9	73.8 ± 6.9	20.7 ± 1.4
HCV Infection						
uPA/SCID	M	4	12	7.5 ± 2.1	74.3 ± 6.8	19.4 ± 1.9
#1C2 homo	M	5	11	12.9 ± 2.2	83.8 ± 5.0	19.7 ± 2.1
#1C2 hemi	M	5	14	10.5 ± 1.1	77.6 ± 3.8	21.5 ± 1.7

## Discussion

We previously reported the successful mass production of chimeric mice with humanized livers using uPA/SCID mice [10]. Notably, the uPA/SCID mice were observed to have intestinal hemorrhagic diathesis (due to high uPA activity), small body size, and kidney disease [13] as well as decreasing h-hep RIs in their livers that was likely due to homologous recombination. In addition, many of the growing m-hep colonies appeared to develop into hepatocellular carcinoma [23]. However, in the present study, we have succeeded in producing mice of this genotype that have overcome all these problems. Furthermore, to our knowledge, this is the first time chimeric mice with humanized livers have been successfully produced using uPA-hemizygous host mice

At first we tried to make a transgenic mouse with cDNA-uPA by the standard protocol, pronuclear microinjection. However, we failed to obtain any mice expressing an adequate level of uPA. We hypothesized that there would be an optimal expression level as host mouse strain for recapitulated with h-heps. Then we changed the protocol to another technique using mouse ES cells. By this protocol we could obtain many more transgenic mouse lines and could evaluate them by their serum ALT activity, a critical criteria of hepatic damage. Of those transgenic mouse lines, 3 lines (#1C2, #2C7, and #1D3) have significant elevations of serum ALT levels. Finally and, to the best of our knowledge, for the first time, we have succeeded in establishing a line, #1C2, as the best host line for making h-hep chimeric mice, because highly repopulated h-heps were observed not only in homozygous but also in hemizygous transgenic mice after being transplanted with h-heps.

Serum uPA activity 3 weeks after birth in uPA/SCID mice was more than 10^4^ times higher than that in #1C2 mice. The reason for this uPA activity difference might be the difference in the copy number of transgenes integrated in the genome. In fact, 5 copies of transgenes were introduced in tandem into the genome of the uPA/SCID mice, while only a single cDNA-uPA transgene was introduced in #1C2 mice. However, ALT levels were almost identical at 3 weeks old, and increased to similar levels at 6 weeks old in both uPA/SCID and cDNA-uPA #1C2 mice. Engraftment of h-heps increased as uPA activity increased, and it appeared that a certain threshold of uPA activity might be necessary for h-heps engraftment into mouse liver. However, h-heps did not engraft into hemizygotes of uPA-NOG mice, which had ALT activity of <100 U/L in 6-week-old mice and then decreased in 8- and 14-week-old mice. We suspect that in #2C7 and uPA-NOG hemizygotes, uPA activity was below the necessary threshold for engraftment of h-heps.

The background of the founder uPA/SCID mice consists of approximately 70–75% c.b.-17, while the remaining 25–30% was determined to originate from the SJL and B6 genetic backgrounds, as indicated by microsatellite analysis (data not shown). Notably, we previously attempted to produce mouse uPA genomic gene-incorporated c.b.-17/SCID congenic mice using the speed congenic method. However, the mice were weak, and all mice transplanted with human hepatocytes died during the analysis. While we were unable to perform a full investigation using these mice, these experiments indicated that a mixed background is essential in the production of chimeric mice using uPA/SCID mice. Thus, in the present study, we have backcrossed cDNA-uPA/SCID mice with SCID-bg mice (a c.b.-17 background of 100%) for two generations in order to introduce the SCID gene. The offspring of this backcross were then mated to uPA (wt/wt)/SCID (+/+) mice (a c.b.-17 background of 70–75%) for two generations. Therefore, the background of the cDNA-uPA/SCID mice analyzed here would have theoretically consisted of 70–75% c.b.-17 background, with the remaining 25–30% being a mix of SJL, B6, and 129SvEv genetic backgrounds.

Since uPA/SCID chimeric mice weighed approximately 18 g and were smaller than wild SCID mice, larger mice would be technically convenient for sequential blood sampling in metabolism studies. However, #1C2 hemizygotes maintained their body weight over a long period, being 4 g heavier than uPA/SCID and #1C2 homozygous mice at around 28 weeks after birth. A notable characteristic was that the mean h-alb level in #1C2 hemizygotes was 15.7 mg/mL, which is approximately 2.9- and 1.6-fold higher than that of uPA/SCID and homozygote chimeric mice, respectively. Based on these results, we concluded that #1C2 hemizygotes were suitable for sequential blood sampling and long-term studies.

Previously reported histopathological findings clearly showed that uPA/SCID chimeric mouse livers were composed of h-hep and degenerative diseased m-hep areas, and uPA-transgene deleted m-hep colonies [2]. We found that #1C2 homo- and hemizygous chimeric mouse livers showed two types of hepatocytes, a h-hep area and a diseased m-hep area, in which many m-heps were cytomegalic, probably because of their capacity for DNA synthesis and incapacity for cell division.

uPA/SCID mice are known to develop kidney disease [13] with enlargement of glomerulus or glomerulosclerosis observed in the kidneys of uPA/SCID chimeric mice. Although these changes, including a defective kidney, were observed in uPA/SCID host mice without transplantation, they were not seen in #1C2 homo- or hemizygotes, and we suspect they were the result of high uPA activity in fetal or neonatal mice blood.

Microarray analyses of chimeric mouse livers revealed that gene expression level correlation coefficients between 2 different hepatocyte donors (BD195, BD72 or BD85) and those of uPA/SCID transplanted mice, ranged from 0.955 to 0.984. Moreover, gene expression correlation coefficients between 2 different host mice, uPA/SCID and #1C2 homozygotes or hemizygotes, transplanted with BD195 cells ranged from 0.985 to 0.997. Because gene expression between a single hepatocyte donor transplanted into a single mouse host is highly correlated (r^2^ > 0.980), hepatocyte gene expressions in chimeric mice were almost identical when the hepatocyte donor was the same, even if the host mice differed. In addition, correlation curves between h-alb and RI were similar among uPA/SCID mice and #1C2 homozygous and hemizygous mice, showing that h-alb synthesis and secretions from h-heps were similar even when transplanted in different host mice. Gene expression levels of all phase I and II drug-metabolizing enzymes remained within a 4-fold difference, and HBV and HCV infectivity was similar among chimeric mice in which BD195 hepatocytes were transplanted using 3 different host mice. HBV copy number in serum was higher in #1C2 homozygous chimeric mice than that in uPA/SCID or #1C2 heterozygous chimeric mice until 40 days after inoculation, although plateau levels of them were similar. Because number of animals used in the studies was limited and h-alb levels were various between groups, susceptibility of chimeric mice using 3 different host mice to HBV or HCV should be determined precisely using larger numbers of animals.

Mating of #1C2 female homozygotes is difficult because #1C2 homozygote females show vaginal and uterus atrophy similar to that of uPA/SCID females [15]. However, an advantage of using #1C2 hemizygotes as hosts for chimeric mice was that host mice (#1C2 hemizygotes) can be obtained by mating #1C2 male homozygotes with female SCID mice, although it is necessary to transplant double the amount of h-heps in order to obtain the same level of engraftment as the homozygotes.

In conclusion, #1C2 homozygotes and hemizygotes are the more suitable host mice to produce chimeric mice containing h-heps and will facilitate long-term studies that require higher RIs.
